# Cost-Effectiveness of Breast Cancer Screening Using Digital Mammography in Canada

**DOI:** 10.1001/jamanetworkopen.2024.52821

**Published:** 2025-01-02

**Authors:** Anna N. Wilkinson, James G. Mainprize, Martin J. Yaffe, Jessica Robinson, Erin Cordeiro, Nicole J. Look Hong, Phillip Williams, Nikitha Moideen, Julie Renaud, Jean M. Seely, Moira Rushton

**Affiliations:** 1 Family Medicine, University of Ottawa Faculty of Medicine, Ottawa, Ontario, Canada; 2 Physical Sciences, Sunnybrook Research Institute, University of Toronto, Toronto, Ontario, Canada; 3 Pharmacy, The Ottawa Hospital Cancer Centre, Ottawa, Ontario, Canada; 4 Department of Surgery, University of Ottawa, Ottawa, Canada; 5 Surgery, University of Toronto St. George Campus, Toronto, Ontario, Canada; 6 Anatomic Pathology, The Ottawa Hospital General Campus, Ottawa, Ontario, Canada; 7 Radiation Oncology, The Ottawa Hospital Cancer Centre, Ottawa, Ontario, Canada; 8 Systemic Therapy, The Ottawa Hospital Cancer Centre, Ottawa, Ontario, Canada; 9 Radiology, University of Ottawa Faculty of Medicine, Ottawa, Ontario, Canada; 10 Medical Oncology, The Ottawa Hospital Cancer Centre, University of Ottawa Faculty of Medicine, Ottawa, Ontario, Canada

## Abstract

**Importance:**

Evolving breast cancer treatments have led to improved outcomes but carry a substantial financial burden. The association of treatment costs with the cost-effectiveness of screening mammography is unknown.

**Objective:**

To determine the cost-effectiveness of population-based breast cancer screening in the context of current treatment standards.

**Design, Setting, and Participants:**

In this economic evaluation, the Canadian Partnership Against Cancer/Statistics Canada OncoSim-Breast microsimulation model was used to estimate the impact of various screening schedules in terms of clinical outcomes and treatment costs. Breast cancer treatment costs were derived from activity-based costing published in 2023 specific to a publicly funded health system in Ontario, Canada. A single birth cohort of individuals assigned female at birth in 1975 was modeled until death or age 99 years (whichever came first).

**Exposures:**

Five screening scenarios were modeled: no screening, biennial (ages 50-74 years and 40-74 years), hybrid (biennial ages 40-49 years and annual ages 50-74 years), and annual screening (ages 40-74 years).

**Main Outcomes and Measures:**

Incremental cost-effectiveness ratios for deaths averted, life-years (LYs) gained, and incremental cost-utility ratios for quality-adjusted life-years (QALYs) gained were determined for screening scenarios. Sensitivity analyses were conducted by varying screening participation rates and reducing recall rates to 5% and the estimated mortality benefits of screening.

**Results:**

Earlier initiation of breast cancer screening at age 40 years (vs age 50 years) was associated with improved clinical outcomes (deaths averted, LYs saved, and QALYs gained) and reduced health care spending on breast cancer treatment. From a health system perspective, incremental cost-effectiveness ratios for biennial screening at ages 40 to 74 years compared with biennial screening at ages 50 to 74 years were cost saving, with CAD$49 759 saved per death averted, $1558 per LY saved, and $2007 saved per QALY gained. Annual screening at ages 40 to 74 years was cost-effective while achieving the best breast cancer outcomes, with costs of $25 501 per death averted, $1100 per LY saved, and $1447 per QALY gained compared with the current Canadian standard of biennial screening at ages 50 to 74 years.

**Conclusions and Relevance:**

In this economic analysis, although screening costs increased according to the number of lifetime screens, they were completely or largely offset by reduced breast cancer therapy costs. Digital mammography was a highly cost-effective tool to reduce breast cancer mortality. These results have important policy implications for all single-payer health systems and call for greater investment in screening programs.

## Key Points

**Question:**

What is the cost-effectiveness of digital mammographic screening for breast cancer?

**Findings:**

In this economic evaluation, biennial screening at ages 40 to 74 years was a cost-saving public health intervention, with CAD$49 759 saved per death averted, $1558 saved per life-year saved, and $2007 saved per quality-adjusted life-year gained, compared with biennial screening at ages 50 to 74 years. Annual screening at ages 40 to 74 years was cost-effective and achieved the best breast cancer outcomes, with $25 501 per death averted, $1100 per life-year saved, and $1447 per quality-adjusted life-year gained, compared with biennial screening at ages 50 to 74 years.

**Meaning:**

These findings suggest that, in addition to averting deaths and saving life-years, earlier initiation of screening mammography at age 40 years yields substantial health care savings by reducing the cost of breast cancer treatment.

## Teaser

This economic evaluation examines the cost-effectiveness of digital mammographic screening for breast cancer in the context of current treatment standards in Canada.

## Introduction

Breast cancer (BC) is the most commonly diagnosed cancer among women and second most common cause of cancer death in North America.^1,2^ Population level mammographic screening effectively decreases mortality and morbidity by diagnosing BC at earlier stages when prognosis is better and treatments less intensive.^3^ Despite the benefits of screening mammography, there is continued public debate about the value of organized screening.^4^

In April 2024, the US Preventive Services Task Force (USPSTF) recommended that BC screening initiation should be lowered from age 50 to 40 years.^5^ The USPSTF acknowledged the increasing incidence of BC in young women,^6^ the mortality benefit of screening,^7^ and the earlier age of diagnosis in women from minoritized racial and ethnic groups.^8^ In Canada, many provinces and territories have screening programs starting at age 40 years^9^; however, the Canadian Task Force on Preventive Health Care does not recommend systematic screening for BC for individuals aged 40 to 49 years at average risk.^10^ Although the USPSTF and Canadian Task Force on Preventive Health Care differ on their recommendations, neither included economic analyses in their guidelines.

BC treatment costs have increased markedly with the introduction of novel therapies in the adjuvant and metastatic settings.^11,12,13,14^ Our group published a per-case, molecular subtype–specific, activity-based costing analysis for BC treatment with 2023 Canadian evidence-based treatment standards.^15^ The costs of treating subtypes of BC (hormone receptor [HR] positive, triple negative, and human epidermal growth factor receptor 2 [ERBB2, previously HER2/neu] positive) increased exponentially with stage, from $14 505 for ductal carcinoma in situ (or stage 0) to $516 415 for HR-positive, ERBB2-positive stage IV BC. In this article, all costs are given in 2023 Canadian dollars.

Previous cost-effectiveness analyses of BC screening have generally found an incremental cost-effectiveness ratio (ICER) within willingness to pay.^16,17^ BC treatment standards have evolved rapidly since prior analyses with nuanced subtype-specific treatment approaches utilizing novel systemic therapies. This economic analysis incorporates comprehensive costing of BC management into an established microsimulation model to evaluate the cost-effectiveness of different regimens for BC screening using digital mammography.

## Methods

### Model Overview

OncoSim-Breast version 3.6.2.4 is a microsimulation model that simulates the natural history, progression, and detection of BC for female individuals, allowing estimation of outcomes of screening and application of treatment costs at a population level.^18^ This model was developed by Statistics Canada and was validated against empirical data from the Canadian Cancer Registry and Vital Statistics. OncoSim-Breast simulates a path through an individual’s life, continually evaluating the possibility for initiation of a cancer, its subtype, and its growth rate, with probabilities based on empirical data. The model was operated by applying user-defined scenarios, with variables such as screening regimen and costs of detection and treatment. The probability of symptomatic or screen detection of cancers was evaluated by the model according to the presence of a cancer screening program, average mammogram sensitivity, and cancer size at each time point. The screened cohort was followed-up to age 99 years. Life tables and random number draws were used to determine cause (other than BC) and time of death. Life-years (LYs) gained and deaths averted through screening were calculated on the basis of life tables for BC modeled deaths. No adjustments for effects of immigration and/or emigration were made. Parameters controlling cancer incidence, progression, and screening performance of digital mammography were calibrated to Canadian Cancer Statistics using the default scenario. The model was adjusted for temporal improvements in survival to reflect improvements in treatment efficacy. Summaries of model inputs and data sources are provided in eTable 1 in Supplement 1. Institutional ethics approval and consent were not required because this was a modeling study that did not involve human participants, in accordance with the Ottawa Hospital Research Institute’s policy.^19^ The Consolidated Health Economic Evaluation Reporting Standards (CHEERS) reporting guideline for economic analysis was followed.

### Costs

Management costs of BC by stage and molecular subtype in Ontario, Canada, were used. The method for determining costs has been previously published and reflects evidence-based standard of care across Canada in 2023.^15^ Screening mammography costs included professional, technical, and administrative costs of breast screening in Ontario, Canada (total $86.92 per screening mammography examination).^20,21^ Diagnostic costs for imaging recalls were calculated separately for each screening regimen modeled.

Existing costs within OncoSim were replaced using 2023 costing data to assign an aggregate cost for each subtype and stage of BC. These costs were applied once at initiation of treatment. Cost values were held fixed for other factors, such as age and tumor grade. Local recurrences were assigned as stage III and distant recurrences as stage IV; the corresponding stage treatments were delivered accordingly. The aggregate costs also included subsequent procedures such as re-excision following local recurrence, diagnostics, and end-of-life care.

### Population

A single birth cohort of individuals born biologically female in 1975 (ie, turning 40 years old in 2015) in Canada was simulated. As of 2015, the cohort consisted of 1 228 636 individuals. The large cohort size was used to reduce statistical fluctuation of results; however, outputs are reported per 1000 women alive in 2015. The model was run for the lifetime of the cohort with outcomes calculated until age 99 years. In these scenarios, all women were screened with digital mammography. Screening regimen was not tailored to breast cancer risk. No differences in cancer risk associated with breast density or ethnicity were included in the scenarios.

### Screening Strategies

Five different digital mammography screening regimens were considered: no screening, biennial screening at ages 50 to 74 years (B50-74), biennial screening at ages 40 to 74 years (B40-74), annual screening at ages 40 to 74 years (A40-74), and a hybrid regimen with annual screening at ages 40 to 49 years and biennial screening at ages 50 to 74 years (A40-49/B50-74). For the base case analysis, 100% participation in each screening regimen was assumed with exactly 12-month or 24-month screening intervals. Ontario screening program recall rates were used.^22^

### Outcomes

For each scenario, the model estimated the number of lifetime screens per person, number of screen-detected BC cases, total number of BC cases, cancer detection rate, recall rate, number of biopsies, stage at BC diagnosis, deaths averted, LYs saved, quality-adjusted life-years (QALYs) gained, and costs of BC management. Total costs of screening, diagnostic workup of imaging recalls, and BC treatment were calculated for each scenario.

### Statistical Analysis

#### Economic Analysis

Discounting was not performed given the anticipated future escalation of systemic therapy costs, which were not included in the modeling. No adjustments were made to reflect priority populations, and there was no patient engagement in the study. Cost-effectiveness analysis included all screening, diagnostic, and treatment costs for any BC diagnosed on or after 2015. Disutilities and utilities were adapted from The Cancer Intervention and Surveillance Modeling Network (CISNET) (eTable 2 in Supplement 1).^23^ Incremental cost-effectiveness ratios (ICERs) and incremental cost-utility ratios (ICURs) were conducted to compare the modeled regimens.

#### Sensitivity Analysis

Sensitivity analyses were performed by scaling data retrospectively subsequent to runs of baseline scenarios. Four alternate sets of conditions were considered. The diagnostic workup costs of screening recalls were doubled. The recall rate was reduced from 9% in the baseline scenarios to the Canadian target of 5% for returning screens.^24^ Benefits (deaths averted and LYs gained) for each scenario were scaled down to the median values presented in the 2024 USPSTF Decision Analysis based on CISNET modeling.^25^ For LY and QALY gains, the USPSTF modeling values were increased by a factor of 1.049, representing the ratio between Canadian and US 2015 life expectancies (83.9 vs 81.3 years).^26,27^ Finally, the association of reduced screening participation with cost outcomes was assessed.

## Results

### Benefits of BC Screening

Results of modeling for each screening regimen can be found in Table 1 and Table 2. The total number of BC cases for all regimens for ages 40 to 99 years varied from 135 to 137. The number of cancers diagnosed during the screening periods varied from 80.3 to 96.7.

**Table 1.** Lifetime Model Outcomes for Screening Mammography by Screening Regimen

Model outcomes	Screening regimen			
	Biennial ages 50-74 y	Biennial ages 40-74 y	Annual ages 40-49 y and biennial ages 50-74 y	Annual ages 40-74 y
Screens per person, mean, No.	11.5	16.1	20.2	31.2
Cases (ages 40-99 y), No.^a^	135.4	135.4	135.6	137.2
Cases (screen period only), No.^a^	80.3	93.4	94.1	96.7
Cancers detected by screen, No.^a^	58.0	64.4	67.8	81.0
Cancer detection rate, No. of cases/1000 screens	5.06	4.00	3.36	2.60
Lifetime recalls, No.^a^	1021	1484	1904	2766
Recall rate, %				
First screen only	19.0	17.5	17.6	17.6
Subsequent rounds	8.0	8.7	9.2	8.7
Overall mean	8.9	9.2	9.4	8.9
Biopsies, No.^a^	143.8	190.8	231.2	320.0
Nonmalignant biopsies, No.^a^	85.7	126.4	163.4	239.0
Biopsy rate (per 1000 screens)	12.5	11.8	11.5	10.3
Biopsy rate (per screen), %	1.25	1.18	1.15	1.03
Benefits^b^				
Deaths averted, No.^a^	10.2	11.9	12.6	15.3
Life-years saved, No.^a^	151.0	203.5	223.3	268.7
Quality-adjusted life-years gained, No.^a^	108.0	148.7	164.1	197.4

^a^ Data are per 1000 women alive at age 40 years.

^b^ Data are compared with no screening.

**Table 2.** Outcomes and Cost-Effectiveness of Screening Regimens Per 1000 Women Alive at Age 40 Years

Outcome	Costs, $^a^						
	Screening regimen					Comparisons	
	No screening	Biennial ages 50-74 y	Biennial ages 40-74 y	Annual ages 40-49 y and biennial ages 50-74 y	Annual ages 40-74 y	Annual ages 40-74 y vs biennial ages 40-74 y	Annual ages 40-74 y vs biennial ages 50-74 y
Screening cost	NA	1 024 807	1 439 689	1 804 272	2 786 526	NA	NA
Diagnostic cost^b^	NA	86 510	127 498	164 913	241 159	NA	NA
Breast cancer management cost	16 330 551	13 671 067	13 133 428	12 856 899	11 884 112	NA	NA
Total cost	16 330 551	14 782 384	14 700 616	14 826 084	14 911 797	NA	NA
Difference vs no screening	NA	−1 548 166	1 629 935	−1 504 467	−1 418 754	NA	NA
Difference vs biennial screening at ages 50-74 y	NA	NA	−81 769	43 700	129 412	NA	NA
Incremental cost	NA	−1 548 166	−81 769	125 468	85 713	211 181	129 412
Deaths averted, No.	NA	10.21	11.86	12.56	15.29	NA	NA
Incremental deaths averted, No.	NA	10.21	1.64	0.71	2.72	3.43	5.07
ICER (deaths averted)	NA	−151 564	−49 759	177 598	31 455	61 543	25 501
LYs gained, No.	NA	151.04	203.51	223.29	268.67	NA	NA
Incremental LYs gained, No.	NA	151.04	52.47	19.78	45.38	65.16	117.63
ICER (LY saved)	NA	−10 250	−1558	6343	1889	3241	1100
QALYs gained, No.	NA	107.98	148.72	164.13	197.39	NA	NA
Incremental QALYs gained, No.	NA	107.98	40.73	15.42	33.26	48.67	89.40
Incremental cost-utility ratio (QALYs gained)	NA	−14 337	−2007	8139	2577	4339	1447

Abbreviations: ICER, incremental cost-effectiveness ratio; LY, life-year; NA, not applicable; QALY, quality-adjusted life-year.

^a^ All costs are shown in 2023 Canadian dollars.

^b^ Diagnostic costs are for recall (no-cancer) workup only. Diagnostic costs for cancer cases are included in the aggregate per-subtype management cost.

Screening B50-74 (the current standard in Canada) found 58 cases (per 1000 women at age 40 years) diagnosed in the modeled population with a mean of 11.5 screening mammograms per person, 1021 recall tests (8.9% recall rate per screen), and 85.7 benign biopsies per 1000 women alive at age 40 years for their remaining lifetime (Table 1). Screening A40-74 resulted in a mean of 31.2 screens per person and detected 81.0 BC cases. A40-74 had a higher absolute number of imaging recalls (2766 recalls) but the same recall rate per screen (8.9%); 153.3 additional benign biopsies were required in this group vs B50-74. Overall, the biopsy rate per 1000 screens was lower for A40-74 compared with B50-74 (10.3 vs 12.5 biopsies per 1000 screens).

**Figure 1.**
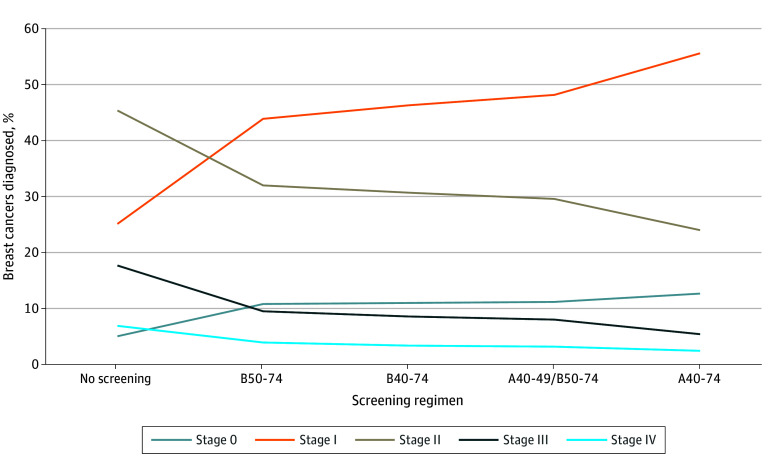
Stage at Which Breast Cancers Were Diagnosed, by Screening Scenarios Stage 0 refers to ductal carcinoma in situ. A40-74 indicates annual screening at ages 40 to 74 years; A40-49/B50-74, annual screening at ages 40 to 49 years and biennial screening at ages 50 to 74 years; B40-74, biennial screening at ages 40 to 74 years; and B50-74, biennial screening at ages 50 to 74 years.

Compared with no screening, there was a lower proportion of stage II, III, and IV BCs and higher rates of stage 0 and stage I BCs (Figure 1 and eTable 3 in Supplement 1). Stage 0 rates increased from 5.0% to 12.6% with A40-74. The proportion diagnosed at stage I increased from 25.0% with no screening to 55.7% with A40-74, with corresponding decreases in stage II (24.0%), III (5.3%), and IV (2.4%) BCs. Comparing across screening strategies, A40-74 shifted the stage at diagnosis down compared with B50-74, with 7 fewer stage II cases, 4 fewer stage III cases, and 1.5 fewer stage IV cases diagnosed per 1000 women alive at age 40 years with 2.1 and 13.5 more stage 0 and I BC cases, respectively.

**Figure 2.**
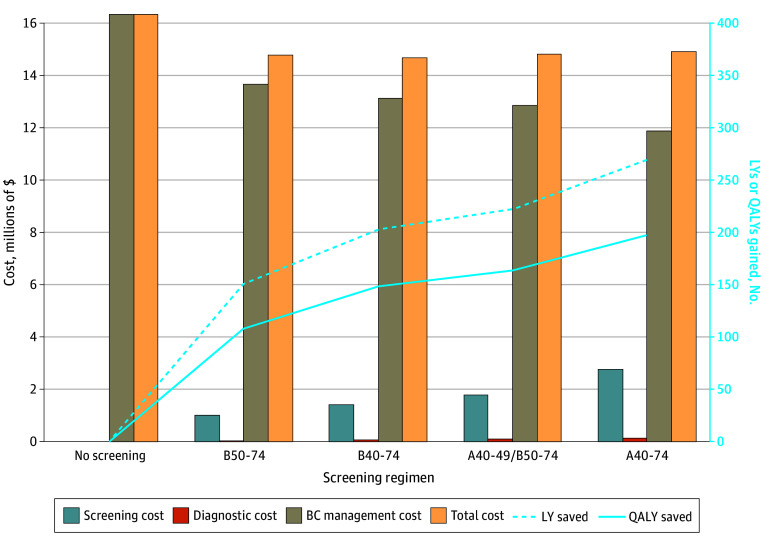
Association of Modeled Screening Mammography Regimens With Costs of Breast Cancer (BC) Screening, Diagnosis, and Management and With Benefits Including Life-Years (LYs) Saved and Quality-Adjusted Life-Years (QALYs) Gained A40-74 indicates annual screening at ages 40 to 74 years; A40-49/B50-74, annual screening at ages 40 to 49 years and biennial screening at ages 50 to 74 years; B40-74, biennial screening at ages 40 to 74 years; and B50-74, biennial screening at ages 50 to 74 years.

Increasing the number of lifetime screens increased the number of deaths averted, LYs saved, and QALYs gained (Table 2 and Figure 2). B50-74 averted 10.2 deaths per 1000 women screened, whereas A40-74 averted 15.3 deaths. LYs saved and QALYs gained also increased along with an increased frequency of screening, with the greatest benefit realized with A40-74 with 268.7 LYs saved and 197.4 QALYs gained per 1000 women alive at age 40 years.

### Population Costs of BC Screening and Treatment

The lifetime cost of screening per 1000 women varied from $0 (no screening) to $2 786 526 for A40-74 (Table 2 and Figure 2). Costs for the workup of imaging recalls were highest in the A40-74 cohort at $241 159. The costs associated with BC treatment were inversely associated with screening, with treatment costs decreasing with more intensive screening regimens. Without BC screening, the lifetime health system cost per 1000 women was $16 330 551 for treatment of BC. The lowest costs of BC treatment were seen with A40-74 at $11 884 112 (Figure 2). Total costs of BC management (screening, recalls, and treatment) varied from $16 330 551 (no screening) to $14 782 384 (B50-74), $14 700 616 (B40-74), $14 826 084 (A40-49/B50-74), and $14 911 797 (A40-74).

### Cost-Effectiveness Analysis

ICERs (cost per death averted, or cost per LY saved) and ICURs (cost per QALY gained) were calculated for each screening regimen (Table 2). Compared with no screening, screening B50-74 and B40-74 were cost-saving to the health system, with associated negative ICERs per ICURs. B40-74 saved the health system $49 759 per death averted, $1558 per LY saved, and $2007 saved per QALY gained.

Incremental benefits of the hybrid strategy were modest, leading to higher ICERs (eg, $177 598 per death averted), indicating that the approach was weakly dominated, and so was not used for comparison with A40-74. The most clinically effective strategy (A40-74) showed acceptable ICERs, with $61 543 per death averted, $3241 per LY saved, and $4339 per QALY gained vs B40-74. Compared with B50-74, A40-74 was even more cost-effective, with $25 501 per death averted, $1100 per LY saved, and $1447 per QALY gained.

### Sensitivity Analyses

When diagnostic workup costs were doubled, the costs per 1000 women increased by $127 498 for B40-74, representing a 0.86% increase in total cost (eTable 4 in Supplement 1); however, a cost savings of $1001 per QALY remained. Total costs for A40-74 increased by 1.59% with an ICER of $4870 per QALY. In the second analysis, cost savings for B40-74 compared with B50-74 increased when imaging recall rates were decreased to the Canadian target of 5%, saving the health system $102 135 per 1000 women alive at age 40 years (eTable 5 in Supplement 1); associated ratios for B40-74 were −$62 153 per death averted, −$1946 per LY saved, and −$2507 per QALY. The hybrid model remained weakly dominated. A40-74 was associated with an ICER of $47 854 per death averted, $2520 per LY saved, and $3374 per QALY gained compared with B40-74.

When examining cost-effectiveness using median outcomes from the USPSTF modeling with a lower number of deaths averted and LY gained,^25^ cancer treatment costs were increased owing to increased costs of treating distant recurrences. Despite the increased costs of cancer management, screening B40-74 remained cost saving ($23 217 saved per 1000 women screened) with ICERs of −$16 729 saved per death averted, −$399 per LY saved, and −$581 saved per QALY gained (eTable 6 in Supplement 1). Here, the hybrid scenario was cost-effective, and for A40-74 the ICER was $370 314 per death averted, with an ICUR of $26 469 per QALY.

**Figure 3.**
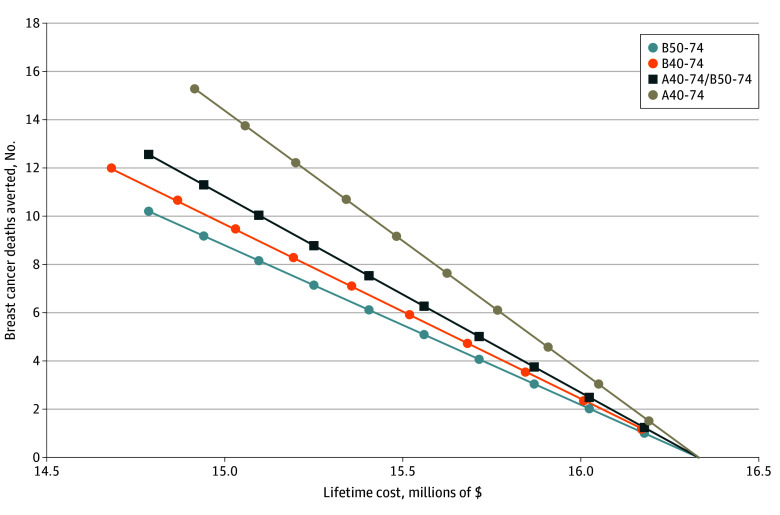
Screening Participation Association With Breast Cancer Outcomes Per 1000 Women Including Death Averted and Lifetime Costs by Screening Regimen A40-74 indicates annual screening at ages 40 to 74 years; A40-74/B50-74, annual screening at ages 40 to 74 years and biennial screening at ages 50 to 74 years; B40-74, biennial screening at ages 40 to 74 years; and B50-74, biennial screening at ages 50 to 74 years.

By varying participation rates for each screening scenario from 0% to 100% in decrements of 10%, we examined the effect of participation on total health system costs and deaths averted (Figure 3). At participation rates of 10% and below, all screening regimens performed similarly. However, 70% participation in A40-74 averted as many BC deaths as approximately 100% participation in B50-74 screening. To achieve the same number of deaths averted as was seen with B50-74 at target participation (70%), less than 50% participation in annual screening would be required. Cost savings were achieved with B40-74 with anything above 30% participation rate.

## Discussion

The treatment of BC has advanced greatly over the past decade, leading to improved clinical outcomes with a considerable financial cost. In this economic evaluation, we assessed cost-effectiveness of mammographic screening based on 2023 evidence-based clinical practices for BC treatment in Canada. Our modeling found that BC screening results in improved clinical outcomes, while enabling reduced treatment costs, at the expense of increased imaging recalls and biopsies. With lifetime follow-up, the total number of cancers accrued is almost completely independent of screening regimen, suggesting minimal overdiagnosis. Depending on the regimen, screening can provide either net savings to the health system or a very modest increase in total cost for the most clinically effective regimen. Screening mammography can achieve a rare outcome for a health intervention: simultaneously improving clinical outcomes and reducing health care costs.

The greatest cost savings were seen with B40-74, whereas the most clinically effective screening strategy was A40-74, which is highly cost-effective by international standards. Target screening participation goals are typically 70%,^28^ whereas actual screening participation may fall below this. The efficacy of A40-74 is such that a 70% annual participation rate would still avert a greater number of deaths than 100% participation in B50-74. Annual screening may present a means to overcome suboptimal participation in screening programs. Improved quality assurance to assist in achieving target recall rates, especially in yearly screening, would further increase cost-effectiveness as noted in our sensitivity analysis.

Multiple studies have found BC screening to be cost-effective using the commonly accepted willingness to pay threshold of $50 000 to $100 000 per QALY.^29^ However, to date, no analyses for average risk screening have shown health system savings. In a systematic review that included 19 studies published from 2015 to 2019 in Europe and North America, B50-74 was most cost efficient, and A40-49/B50-74 was within a willingness to pay of $100 000 per QALY.^17^ Cost-effectiveness increased when BC screening was risk-based. An Australian study found an ICER of $40 279 (2018 Australian dollars) per LY gained,^30^ whereas a European review of 32 screening studies found gains in life expectancy in all, with an ICER less than €30,000 (2017 euros) per LY gained.^31^ Compared with no screening, the CISNET model found improved QALYs and decreased BC mortality with screening, with an ICUR of $50 223 (2017 US dollars) per QALY gained.^32^ Looking specifically at screening in younger women in the US, annual screening beginning at age 45 years with biennial screening from ages 55 to 75 years was most cost-effective at an ICER of $40 135 (2017 US dollars) per QALY.^33^ A previous OncoSim-Breast model with 2012 costs found an ICUR of $38 142 (2012 Canadian dollars) per QALY for biennial screening at ages 50 to 69 years and $83 845 (2012 Canadian dollars) per QALY for A40-74.^16^

Our results differ from previous cost-effectiveness analyses primarily because of recent innovations in systemic therapy. Systemic therapy costs account for up to 9.6 times the cost of all other components of BC therapy combined.^15^ CDK4/6 inhibitors were introduced in 2016 for metastatic HR-positive BC and in 2022 for adjuvant treatment of high-risk, HR-positive BC.^34^ These drugs have a substantial financial burden, as abemaciclib costs $141 796 for 2 years in the adjuvant setting (stage III) and $210 926 if given for 3 years in the metastatic setting.^35,36^ The antibody-drug conjugate trastuzumab deruxtecan (approved in 2022) for metastatic ERBB2-positive and ERBB2-low BC comes at a cost of $165 949 for 1 year of therapy, whereas the antibody-drug conjugate sacituzumab govitecan (approved in 2023) for metastatic triple-negative BC costs $204 945 for 1 year of treatment.^37,38^ Immunotherapy for stage II to IV triple-negative BC (funded in 2023) costs $152 529 for 1 year of therapy.^39^ These new therapies are highly effective and have improved outcomes for patients with BC, meaning that women with stage IV BC are living longer and incurring treatment costs over a longer duration of time. The median overall survival today for stage IV HR-positive or ERBB2-positive BC is over 5 years.^40^ Previous costing analyses looked only at BC treatment costs over a defined time frame, often 2 years, while activity-based costing captures the entire cost for the treatment for each case of BC, independent of duration.^41^

### Limitations

There are several limitations of this study. An average sensitivity was used for mammography, which may not adequately capture impacts of breast density on screening efficacy. Recall rates are consistent with those reported for Ontario in 2023,^22^ but do not meet the Canadian target of 5%. Implementation of artificial intelligence to decrease recall rates in the future may improve cost-effectiveness further.^42^ Despite comprehensive costing of BC treatment, there are costs that we could not include. We did not include health costs outside the public system (eg, private drug costs or private allied health supports). The costs of complications of BC and treatment (eg, febrile neutropenia, venous thromboembolic disease, and pathological fractures) were conservatively estimated, which would preferentially impact costs for advanced-stage disease. We also did not include the societal costs of BC, such as lost productivity owing to time off work and inability to fulfill caregiver duties during treatment. Morbidity of BC treatment is estimated using utilities for investigations and treatments applied at each stage. Although cost, duration, and intensity of systemic therapies have changed greatly, utility assessments for modeling of BC outcomes have not kept pace, so that the true morbidity of BC treatment, and, therefore, the benefit of early detection is not adequately captured. The cost-effectiveness of other screening modalities such as tomosynthesis, magnetic resonance imaging, and supplemental screening for dense breasts, as well as risk-stratified screening approaches, are other important areas to investigate in the future. Primary prevention, including modification of lifestyle factors, could potentially provide further cost savings to BC treatment.

## Conclusions

As costs of BC management have increased with advances in treatment, so has the cost-effectiveness of early detection with mammographic screening when compared with older analyses. Despite conservative estimates for advanced stage disease and no consideration for societal costs, our model demonstrates cost-effectiveness of annual mammography screening at ages 40 to 74 years for women at average risk. We also show the 2024 USPSTF recommended strategy of biennial screening from ages 40 to 74 years is cost-savings from the health system perspective. This work highlights the importance of public health and funders regularly assessing updated cost-effectiveness when evaluating the risks and benefits of population-based screening programs. The cost of cancer management is likely going to continue to increase^14^; accordingly, BC diagnosis at earlier stages, when management costs are less, will be increasingly important. Organized BC screening programs enable earlier detection of BC, improving clinical outcomes while simultaneously minimizing treatment burden and costs, thereby improving health equity.

## Notes

Supplement 1. **eTable 1.** OncoSim-Breast model inputs and data source descriptions **eTable 2.** (Dis)Utilities adapted from The Cancer Intervention and Surveillance Modeling Network (CISNET) **eTable 3.** Stage and subtype distribution cancers diagnosed in each tested screening scenario **eTable 4.** Sensitivity analysis with diagnostic workup costs for recalled participants (no cancer) increased by a factor of 2 **eTable 5.** Sensitivity analysis with benefits (deaths averted, LY gained) for abnormal recall rate reduced to target of 5% for recurrent screening. **eTable 6.** Sensitivity analysis with benefits (deaths averted, LY gained) for each scenario scaled to the median values in the 2024 USPSTF Decision Analysis based on CISNET modeling

Supplement 2.
Data Sharing Statement
