# Supplementary material for: Cost-Effectiveness of Breast Cancer Screening Using Digital Mammography in Canada
Source: JAMA Netw Open. 2025 Jan 2;8(1):e2452821. doi: 10.1001/jamanetworkopen.2024.52821 (PMC11696453; doi:10.1001/jamanetworkopen.2024.52821)
Supplement: Supplement 1. — eTable 1. OncoSim-Breast model inputs and data source descriptions eTable 2. (Dis)Utilities adapted from The Cancer Intervention and Surveillance Modeling Network (CISNET) eTable 3. Stage and subtype distribution cancers diagnosed in each tested screening scenario eTable 4. Sensitivity analysis with diagnostic workup costs for recalled participants (no cancer) increased by a factor of 2 eTable 5. Sensitivity analysis with benefits (deaths averted, LY gained) for abnormal recall rate reduced to target of 5% for recurrent screening. eTable 6. Sensitivity analysis with benefits (deaths averted, LY gained) for each scenario scaled to the median values in the 2024 USPSTF Decision Analysis based on CISNET modeling [file jamanetwopen-e2452821-s001.pdf]

Supplemental Online Content

Wilkinson AN, Mainprize JG, Yaffe MJ, et al. Cost-effectiveness of breast cancer screening using digital mammography in Canada. *JAMA Netw Open*. 2025;8(1):e2452821. doi:10.1001/jamanetworkopen.2024.52821

eTable 1. OncoSim-Breast model inputs and data source descriptions

eTable 2. (Dis)Utilities adapted from The Cancer Intervention and Surveillance Modeling Network (CISNET)

eTable 3. Stage and subtype distribution cancers diagnosed in each tested screening scenario

eTable 4. Sensitivity analysis with diagnostic workup costs for recalled participants (no cancer) increased by a factor of 2

eTable 5. Sensitivity analysis with benefits (deaths averted, LY gained) for abnormal recall rate reduced to target of 5% for recurrent screening.

eTable 6. Sensitivity analysis with benefits (deaths averted, LY gained) for each scenario scaled to the median values in the 2024 USPSTF Decision Analysis based on CISNET modeling

This supplemental material has been provided by the authors to give readers additional information about their work.

© 2025 Wilkinson AN et al. *JAMA Network Open.*

eTable 1 OncoSim-Breast model inputs and data source descriptions

Model input	Updates
Population demography	Includes more recent observed data (up to 2019 for birth, immigration, and interprovincial migration; up to 2021 for all-cause mortality) and projections from Statistics Canada.
Oncogenesis (rate of tumour onset)	Calibration target includes the latest available incidence data in the Canadian Cancer Registry (observed data up to 2020).
Stage-specific Survival	Calibrated to match the latest published data from the Canadian Cancer Registry (2017).
Mammogram specificity	Calibrated to match more recent abnormal call rates data from screening programs (2019).
Breast cancer costs	Updated costs for stage and subtype from Wilkinson et al. (2023)
Decrement of health state utility due to breast cancer	Aligned data and assumptions with other established breast cancer models (CISNET-Breast).

eTable 2 (Dis)Utilities adapted from The Cancer Intervention and Surveillance Modeling Network (CISNET).[22]

	Utility	Disutility	Duration
Diagnostic workup and staging	0.895	0.105	1 Week
Cancer treatment for Node-Negative <1 cm	0.895	0.105	1 year
Cancer treatment for Node-Negative, >1 cm	0.9	0.1	1 year
Cancer treatment for Node Positive, low burden	0.9	0.1	1 year
Cancer treatment for Stage III	0.75	0.25	1 year
Cancer treatment for Stage IV	0.6	0.4	1 year
End of Life Care	0.179	0.821	3 months

© 2025 Wilkinson AN et al. *JAMA Network Open.*

eTable 3 Stage and subtype distribution cancers diagnosed in each tested screening scenario

Stage	No Screening	Biennial 50-74	Biennial 40-74	Annual 40-49/ Biennial 50-74	Annual 40-74
Stage 0	4.1 (5.0%)	10.3 (10.7%)	10.5 (10.9%)	10.7 (11.1%)	12.4 (12.6%)
Stage I	20.5 (25.0%)	42.2 (44.0%)	44.4 (46.4%)	46.4 (48.2%)	55.0 (55.7%)
Stage II	37.3 (45.5%)	30.7 (31.9%)	29.4 (30.7%)	28.5 (29.6%)	23.7 (24.0%)
Stage III	14.5 (17.7%)	9.2 (9.5%)	8.2 (8.5%)	7.6 (7.9%)	5.2 (5.3%)
Stage IV	5.6 (6.8%)	3.7 (3.9%)	3.3 (3.4%)	3.1 (3.2%)	2.4 (2.4%)
Subtype					
HR+	74.3%	77.4%	77.6%	77.8%	78.9%
HR+/HER2+	10.9%	9.5%	9.5%	9.3%	8.9%
HER2+	5.3%	4.5%	4.3%	4.3%	4.0%
TN	9.5%	8.6%	8.6%	8.5%	8.1%

Numbers expressed per 1000 women alive at age 40. Numbers in parentheses represent the percentage for each stage for a given screening regimen

© 2025 Wilkinson AN et al. *JAMA Network Open.*

eTable 4 Sensitivity analysis with diagnostic workup costs for recalled participants (no cancer) increased by a factor of 2.

	Bienn. 50 – 74	Bienn. 40 - 74	Ann. 40-49 Bienn. 50-74	Ann.40 - 74	Annual 40-74 (compared to B40-74)
**Screening Cost**	$1,024,807	$1,439,689	$1,804,272	$2,786,526	
	-	-	-	-	
**Diagnostic Cost†**	$173,020	$254,997	$329,826	$482,319	
	+$86,510	*+$127,498*	*+$164,913*	*+$241,159*	
**BC Management Cost**	$13,671,067	$13,133,428	$12,856,899	$11,884,112	
	-	-	-	-	
**Total Cost**	$14,868,894	$14,828,114	$14,990,997	$15,152,956	
	+$86,510	*+$127,498*	*+$164,913*	*+$241,159*	
**Cost Difference (No Screening)**	($1,461,657)	($1,502,437)	($1,339,554)	($1,177,595)	
	+$86,510	*+$127,498*	*+$164,913*	*+$241,159*	
**Cost Difference (50-74 Biennial)**	-	($40,780)	$122,103	$284,062	
	-	*+$40,989*	*+$78,403*	*+$154,649*	
**Deaths Averted**	10.21	11.86	12.56	15.29	
	-	-	-	-	
**ICER (Deaths averted)**	($143,095)	($24,816)	$230,558	$59,435	$94,666
	+$8,469	*+$24,943*	*+$52,960*	*+$27,981*	*+$33,123*
**LY Gained**	151.04	203.51	223.29	268.67	0.00
	-	-	-	-	-
**ICER (LY saved)**	($9,677)	($777)	$8,235	$3,569	$4,986
	+$573	*+$781*	*+$1,892*	*+$1,680*	*+$1,744*
**QALY Gained**	107.98	148.72	164.13	197.39	0.00
	-	-	-	-	*+0.00*
**ICUR (QALY Gained)**	($13,536)	($1,001)	$10,566	$4,870	$6,674
	+$801	*+$1,006*	*+$2,427*	*+$2,293*	*+$2,335*

†Diagnostic costs for recall (no-cancer) workup only. Diagnostic costs for cancer cases are included in the aggregate per-subtype management cost

BC: Breast Cancer; LY: Life Years; QALY: Quality Adjusted Life Year; ICER: Incremental Cost-Effectiveness Ratio; ICUR: Incremental Cost-Utility Ratio

Parentheses indicate negative dollar amounts

Numbers in italics indicate the change compared to the baseline scenarios.

© 2025 Wilkinson AN et al. *JAMA Network Open.*

eTable 5. Sensitivity analysis with benefits (deaths averted, LY gained) for abnormal recall rate reduced to target of 5% for recurrent screening.

	**Bienn. 50 – 74**	**Bienn. 40 - 74**	**Ann. 40-49 Bienn. 50-74**	**Ann.40 - 74**	**Ann 40-74 (compared to B40-74)**
**Screening Cost**	$1,024,807	$1,439,689	$1,804,272	$2,786,526	
	-	-	-	-	
**Diagnostic Cost†**	$48,545	$69,168	$87,391	$135,855	
	-$37,965	*-$58,331*	*-$77,522*	*-$105,305*	
**BC Management Cost**	$13,671,067	$13,133,428	$12,856,899	$11,884,112	
	-	-	-	-	
**Total Cost**	$14,744,420	$14,642,285	$14,748,562	$14,806,492	
	-$37,965	*-$58,331*	*-$77,522*	*-$105,305*	
**Cost Difference (No Screening)**	($1,586,131)	($1,688,266)	($1,581,988)	($1,524,059)	
	-$37,965	*-$58,331*	*-$77,522*	*-$105,305*	
**Cost Difference (50-74 Biennial)**	-	($102,135)	$4,143	$62,072	
	-	*-$20,366*	*-$39,557*	*-$67,340*	
**Deaths Averted**	10.21	11.86	12.56	15.29	
	-	-	-	-	
**ICER (Deaths averted)**	($155,281)	($62,153)	$150,434	$21,259	$47,854
	-$3,717	*-$12,394*	*-$27,164*	*-$10,196*	*-$13,689*
**LY Gained**	151.04	203.51	223.29	268.67	
	-	-	-	-	
**ICER (LY saved)**	($10,502)	($1,946)	$5,373	$1,277	$2,520
	-$251	*-$388*	*-$970*	*-$612*	*-$721*
**QALY Gained**	107.98	148.72	164.13	197.39	
	-	-	-	-	
**ICUR (QALY Gained)**	($14,689)	($2,507)	$6,894	$1,742	$3,374
	-$352	*-$500*	*-$1,245*	*-$835*	*-$965*

†Diagnostic costs for recall (no-cancer) workup only. Diagnostic costs for cancer cases are included in the aggregate per-subtype management cost

BC: Breast Cancer; LY: Life Years; QALY: Quality Adjusted Life Year; ICER: Incremental Cost-Effectiveness Ratio; ICUR: Incremental Cost-Utility Ratio

Parentheses indicate negative dollar amounts

Numbers in italics indicate the change compared to the baseline scenarios.

© 2025 Wilkinson AN et al. *JAMA Network Open.*

eTable 6 Sensitivity analysis with benefits (deaths averted, LY gained) for each scenario scaled to the median values in the 2024 USPSTF Decision Analysis based on CISNET modeling.[24]

	**Bienn. 50 - 74**	**Bienn. 40 - 74**	**Ann. 40-49 Bienn. 50-74**	**Ann.40 - 74**	**Ann 40-74 (compared to B40-74)**
**Screening Cost**	$1,024,807	$1,439,689	$1,804,272	$2,786,526	
	-	-	-	-	
**Diagnostic Cost †**	$86,510	$127,498	$164,913	$241,159	
	-	-	-	-	
**BC Management Cost**	$14,126,981	$13,647,894	$13,277,276	$12,657,492	
	+$455,914	*+$514,466*	*+$420,377*	*+$773,380*	
**Total Cost**	$15,238,298	$15,215,081	$15,246,461	$15,685,177	
	+$455,914	*+$514,466*	*+$420,377*	*+$773,380*	
**Cost Difference (No Screening)**	($1,092,252)	($1,115,469)	($1,084,090)	($645,374)	
	+$455,914	*+$514,466*	*+$420,377*	*+$773,380*	
**Cost Difference (50-74 Biennial)**	-	($23,217)	$8,162	$446,878	
	-	*+$58,552*	*-$35,537*	*+$317,466*	
**Deaths Averted**	8.23	9.61	10.73	11.91	
	-1.99	*-2.24*	*-1.83*	*-3.37*	
**ICER (Deaths averted)**	($132,792)	($16,729)	$28,092	$370,314	$204,236
	+$18,772	*+$33,030*	*-$149,506*	*+$338,860*	*+$142,693*
**LY Gained**	120.22	178.44	187.67	218.93	
	-30.82	*-25.07*	*-35.62*	*-49.73*	
**ICER (LY saved)**	($9,086)	($399)	$3,399	$14,034	$11,609
	+$1,165	*+$1,160*	*-$2,944*	*+$12,145*	*+$8,368*
**QALY Gained**	84.66	124.63	128.19	144.77	
	-23.33	*-24.09*	*-35.94*	*-52.62*	
**ICUR (QALY Gained)**	($12,902)	($581)	$8,798	$26,469	$23,340
	+$1,435	*+$1,427*	*+$659*	*+$23,892*	*+$19,001*

†Diagnostic costs for recall (no-cancer) workup only. Diagnostic costs for cancer cases are included in the aggregate per-subtype management cost

BC: Breast Cancer; LY: Life Years; QALY: Quality Adjusted Life Year; ICER: Incremental Cost-Effectiveness Ratio; ICUR: Incremental Cost-Utility Ratio

Parentheses indicate negative dollar amounts

Numbers in italics indicate the change compared to the baseline scenarios.

© 2025 Wilkinson AN et al. *JAMA Network Open.*
