# Supplementary material for: Cost-Effectiveness of Breast Cancer Screening Using Digital Mammography in Canada
Source: JAMA Netw Open. 2025 Jan 2;8(1):e2452821. doi: 10.1001/jamanetworkopen.2024.52821 (PMC11696453; doi:10.1001/jamanetworkopen.2024.52821)
Supplement: Supplement 2. — Data Sharing Statement [file jamanetwopen-e2452821-s002.pdf]

Data Sharing Statement

Wilkinson. Cost-Effectiveness of Breast Cancer Screening Using Digital Mammography in Canada. *JAMA Netw Open*. Published January 02, 2025.
doi:10.1001/jamanetworkopen.2024.52821

Data
Data available: Yes
Data types: Data (not involving human participants)
How to access data: The authors would be pleased to share data tables for the purposes of academic research with other investigators upon reasonable request. Please contact: martin.yaffe@sri.utoronto.ca. Those wishing access to the OncoSim Breast model should contact the Canadian Partnership Against Cancer directly (https://www.partnershipagainstcancer.ca/tools/oncosim/request-account/).
When available: With publication

Supporting Documents
Document types: None

Additional Information
Who can access the data: The authors would be pleased to share data tables for the purposes of academic research with other investigators upon reasonable request.
Types of analyses: Academic
Mechanisms of data availability: Contact Dr. Yaffe (martin.yaffe@sri.utoronto.ca).
